# A Chemical Proteomics Approach to Discover Regulators of Innate Immune Signaling

**DOI:** 10.3390/v15051112

**Published:** 2023-05-03

**Authors:** Andrew P. Kurland, Boris Bonaventure, Jeffrey R. Johnson

**Affiliations:** 1 Department of Microbiology, Icahn School of Medicine at Mount Sinai, New York, NY 10029, USA; 2 Global Health and Emerging Pathogens Institute, Icahn School of Medicine at Mount Sinai, New York, NY 10029, USA

**Keywords:** proteomics, phosphoproteomics, kinases, interferon, RIG-I, signaling pathways, ATM, ATR, AMPK, PLK1

## Abstract

Innate immune pathways are tightly regulated to balance an appropriate response to infectious agents and tolerable levels of inflammation. Dysregulation of innate immune pathways can lead to severe autoinflammatory disorders or susceptibility to infections. Here, we aimed to identify kinases in common cellular pathways that regulate innate immune pathways by combining small-scale kinase inhibitor screening with quantitative proteomics. We found that inhibitors of kinases ATM, ATR, AMPK, and PLK1 reduced the induction of interferon-stimulated gene expression in response to innate immune pathway activation by poly(I:C) transfection. However, siRNA depletion of these kinases did not validate findings with kinase inhibitors, suggesting that off-target effects may explain their activities. We mapped the effects of kinase inhibitors to various stages in innate immune pathways. Determining the mechanisms by which kinase inhibitors antagonize these pathways may illuminate novel mechanisms of innate immune pathway control.

## 1. Introduction

Human cells have evolved innate immune defenses to recognize and rapidly respond to viruses. Human-encoded pattern recognition receptors (PRRs) recognize pathogen-associated molecular patterns (PAMPs) to trigger signaling pathways that lead to the production of inflammatory cytokines, including interferons (IFNs) [1,2,3]. PRRs detect multiple types of PAMPs, including lipids, proteins, and nucleic acids. The RIG-I-like receptors (RLRs) RIG-I and MDA5 detect aberrant RNA species that indicate the presence of viral infection, such as 5′-triphosphorylated RNA for RIG-I [2]. Upon detection, RIG-I and MDA5 recruit the mitochondrial antiviral-signaling protein (MAVS) to activate a downstream signaling pathway involving the kinases TBK1 and IKKε, leading to the phosphorylation, dimerization, and nuclear translocation of interferon regulatory factor (IRF) transcription factors that lead to production and secretion of type-I IFN. Secreted type-I IFN acts in an autocrine and paracrine manner by binding to cell surface interferon-α/β receptors (IFNARs), which induce signaling via a JAK-STAT pathway. IFNAR activation leads to phosphorylation of STAT1 and STAT2 and association with IRF9 to form the ISGF3 transcription factor complex. This is followed by the nuclear translocation of ISGF3 and ISGF3 binding to interferon-stimulated response elements (ISREs) in the genome, leading to transcriptional activation of hundreds of interferon-stimulated genes (ISGs), among which many are potent and direct antiviral effectors [4,5]. The IFN pathway establishes a complete transcriptional reprogramming hostile to viral replication.

Viruses have evolved mechanisms to antagonize these innate immune pathways. Some examples include the degradation of STAT2 by flaviviruses, the inhibition of IFN mRNA maturation and translation by influenza viruses, and the inhibition of ISGF3 nuclear translocation by SARS-CoV-2 [6,7,8,9,10]. Given their limited coding capacity, viruses frequently rely on manipulating host cellular pathways to effectively antagonize innate immune pathways. Dysregulation of innate immune pathways can lead to inflammatory disease states or enhanced susceptibility to infections. Thus, a better understanding of the factors that regulate innate immune pathways can reveal the mechanisms underlying viral antagonism and innate immune pathway dysregulation in disease states. 

While the canonical components of RLR and IFN signaling are well defined, external factors regulating these pathways are only beginning to be explored with systematic technologies. A recent genome-wide CRISPR screen revealed BRD9 as a critical and druggable factor required for ISG expression and antiviral activity [11]. Here, we combined quantitative proteomics and phosphoproteomics with kinase inhibitor screening to characterize innate immune signaling pathways. We found that RLR and IFN pathway activation results in a reduction in most cellular kinase activities. We also found that pretreatment of cells with inhibitors of ATM, ATR, AMPK, and PLK1 reduced the induction of ISGs in response to poly(I:C) transfection in A549 human lung epithelial cells. Finally, we mapped inhibitor activities to multiple steps in RLR and IFN pathways. These findings suggest novel mechanisms of regulation of innate immune pathways by crosstalk with common cellular pathways.

## 2. Materials and Methods

Cell culture. A549 human lung epithelial cells (A549, ATCC^®^, CCL-185) were cultured in in Dulbecco’s Modified Eagle Medium (Corning, Corning, NY, USA) supplemented with 10% *v*/*v* fetal bovine serum (Thermo Fisher Scientific, Waltham, MA, USA), 100 I.U./mL penicillin, and 100 µg/mL streptomycin (Corning).

Poly(I:C) transfection time course proteomics. A549 cells were transfected with poly(I:C) (HMW, Invivogen, San Diego, CA, USA) using Lipofectamine 3000 reagent (Thermo Scientific, Waltham, MA, USA). One set of plates was lysed prior to transfection for an untransfected negative control. Transfected plates were lysed at 5 min, 30 min, 2 h, and 24 h post-transfection in a buffer comprised of 8 M Urea (Sigma, St. Louis, MO, USA), 50 mM ammonium bicarbonate (Sigma), 150 mM NaCl (Sigma), protease/phosphatase inhibitor cocktail (HALT, Thermo Scientific) prepared in Optima LC/MS grade water (Fisher Scientific, Waltham, MA, USA). Samples were prepared for quantitative proteome and phosphoproteome analysis using a previously described method [12,13].

Inhibitor treatment and poly(I:C) transfection for proteomics. Inhibitors used for this study were obtained from Selleck Chemicals: ERK1/2: Ravoxertinib (GDC-0994, Cat. # S7554), ERK2: Ulixertinib (BVD-523, Cat. # S7854), AURKA: Alisertib (MLN8237, Cat. # S1133), AURKB: Barasertib (AZD1152-HQPA, Cat. # S1147), JNK1/2/3: Tanzisertib (CC-930, Cat. # S8490), p38a: Ralimetinib (LY2228820, Cat. # S1494), p38/b: Losmapimod (GW856553X, Cat. # S7215), ATM: KU-55933 (Cat. # S1092), ATR: VX-803 (M4344, Cat #. S9639), PLK1: Volasertib (BI6727, Cat. #S2235), mTOR: Rapamycin (AY-22989, Cat. # S1039), GSK3A/B: Laduviglusib (CT99021, Cat. # S1263), AMPKA1: Dorsomorphin (Compound C, Cat. # S7840), TBK1/iKKε: MRT67037 HCl (Cat. # S7948).

A549 cells were pretreated with inhibitors at 10 µM or an equal volume of DMSO in biological triplicate. Then, 1 h after inhibitor treatment, cells were transfected with poly(I:C)/Lyovec (HMW, Invivogen) at 0.5 µg/mL concentration. Then, 24 h after poly(I:C) transfection, cells were lysed in 0.025% RapiGest (Waters, Milford, MA, USA), 2 M Urea (Sigma), 50 mM ammonium bicarbonate (Sigma), 150 mM NaCl (Sigma), supplemented with a protease/phosphatase inhibitor cocktail (HALT, Thermo Scientific) and prepared in Optima LC/MS grade water (Fisher Scientific). Samples were prepared for quantitative proteome analysis using a previously described method [12,13].

siRNA transfection and poly(I:C) transfection proteomics. SiGENOME SMARTpool siRNA pools targeting ATM (M-003201-04-0005), ATR (M-003202-05-0005), PLK1 (M-003290-01-0005), AMPKA1 (M-005027-02-0005), and non-targeting control (D-001206-13-20) were obtained from Horizon Discovery. siRNA pools were transfected into A549 cells with lipofectamine RNAiMAX reagent (Thermo Scientific) according manufacturer’s instructions. Then, 48 h after siRNA transfection, cells were transfected with poly(I:C)/Lyovec (HMW, Invivogen) at 0.5 µg/mL concentration. Then, 24 h after poly(I:C) transfection, cells were lysed in 0.025% RapiGest (Waters), 2M Urea (Sigma), 50 mM ammonium bicarbonate (Sigma), 150 mM NaCl (Sigma), in Optima LC/MS grade water (Fisher Scientific). Samples were prepared for proteomics analysis as described previously [12,13].

Cell viability assays. A549 cells were treated with each inhibitor at 10 µM in triplicate. Cell viability assays were performed 24 h post-treatment with the Cell TiterGlo reagent (Promega, Madison, WI, USA) using the manufacturer’s protocol. Luminescence was read with end-point kinetics with a 100 ms integration on a Cytation 5 Plate Reader using Gen5 software (Biotek Instruments, Winooski, VT, USA). Two-tailed paired student’s *t*-tests for unequal variances were performed to generate *p*-values, which were corrected for multiple testing by Bonferroni correction.

Inhibitor and IFN treatment: A549 cells were pretreated with 10 µM inhibitors for PLK1, ATM, ATR, and AMPKA1. After 1 h of treatment cells were treated 1000 I.U./mL IFNβ1a (PBL assay science Cat. # 11410). Then, 24 h after IFN treatment, cells were washed three times in PBS and lysed in 0.025% RapiGest (Waters), 2 M Urea (Sigma), 50 mM ammonium bicarbonate (Sigma), 150 mM NaCl (Sigma), supplemented with protease/phosphatase inhibitor cocktail (HALT, Thermo Scientific) prepared in Optima LC/MS grade water (Fisher Scientific). Samples were diluted in Laemmli buffer for gel electrophoresis and Western blotting.

Antibodies. Antibodies used for Western blotting included anti-MX1 (Cell Signaling, Cat. # 37849), anti-phopsho-STAT1 Y701 (Cell Signaling, Cat. # 9167S), anti-ATM (Cell Signaling, Cat. # 2873T), anti-ATR (Cell Signaling, Cat. # 3934S), anti-AMPKA1 (Cell Signaling, Cat. # 2795T), anti-GAPDH (Cell Signaling, Cat. # 2118S), and goat anti-rabbit-HRP (Bio-Rad, Cat. # 1706515). Primary antibodies were incubated at 4 °C overnight at a 1:1000 dilution in 5% milk in Tris-buffer saline with 0.1% Tween-20 (TBS-T), secondary antibodies were incubated for 1 h at room temperature at a 1:3000 dilution in 5% milk in TBS-T.

Mass spectrometry data acquisition. All samples were analyzed on an Orbitrap Eclipse mass spectrometry system equipped with an Easy nLC 1200 ultra-high pressure liquid chromatography system interfaced via a Nanospray Flex nanoelectrospray source (Thermo Fisher Scientific). Samples were injected onto a column (30 cm × 75 μm (ID), CoAnn Technologies, Richland, WA, USA) packed with ReprosilPur C18-AQ 1.9 μm particles (Dr. Maisch GmbH, Ammerbuch, Germany). Mobile phase A consisted of 0.1% formic acid (FA), and mobile phase B consisted of 0.1% FA/80% acetonitrile (ACN). Peptides were separated by an organic gradient from 5% to 35% mobile phase B over (120 minfor poly(I:C) transfection time course, 60 min for siRNA transfection proteomics, and 30 min for kinase inhibitor treatment proteomics) followed by an increase to 100% B over 10 min at a flow rate of 300 nL/min. Analytical columns were equilibrated with 3 μL of mobile phase A.

To build a spectral library, samples from each set of biological replicates were pooled and acquired in a data-dependent manner. Protein abundance samples for the poly(I:C) transfection time course were fractionated with Field Asymmetric Ion Mobility Spectrometry (FAIMS) fractionation with a FAIMS Pro device (Thermo Fisher Scientific). Each pooled sample was analyzed 4 times with 4 different FAIMS compensation voltages (CV) (−40 V, −55 V, −65 V, −75 V). Data-dependent analysis (DDA) was performed by acquiring a full scan over a *m*/*z* range of 375–1025 in the Orbitrap at 120,000 resolving power (@200 *m*/*z*) with a normalized AGC target of 100%, an RF lens setting of 30%, and an instrument-controlled ion injection time. Dynamic exclusion was set to 30 s, with a 10 p.p.m. exclusion width setting. Peptides with charge states 2–6 were selected for MS/MS interrogation using higher energy collisional dissociation (HCD) with a normalized HCD collision energy of 28%, with 3 s of MS/MS scans per cycle. Similar settings were used for data-dependent analysis (DDA) of phosphopeptide enriched pooled samples, with a dynamic exclusion of 45 s and no FAIMS fractionation.

Data-independent analysis (DIA) was performed on all individual samples. An MS scan at 60,000 resolving power over a scan range of 390–1010 *m*/*z*, an instrument controlled AGC target, an RF lens setting of 30%, and an instrument controlled maximum injection time, followed by DIA scans using 8 *m*/*z* isolation windows over 400–1000 *m*/*z* at a normalized HCD collision energy of 28%.

Mass spectrometry data analysis. The Spectronaut algorithm was used to build spectral libraries from DDA data, identify peptides/proteins, localize phosphorylation sites, and extract intensity information of fragment ions [14]. Data were searched against the human sequences in the SwissProt portion of the UniProt database (downloaded on 10 October 2019). False discovery rates were estimated using a decoy database strategy [15]. All data were filtered to achieve a false discovery rate of 0.01 for peptide-spectrum matches, peptide identifications, and protein identifications. Search parameters included a fixed modification for carbamidomethyl cysteine and variable modifications for N-terminal protein acetylation, methionine oxidation, and for phosphoproteomics samples, serine, threonine, and tyrosine phosphorylation. All other search parameters were Biognosys factory defaults.

Statistical analysis of proteomics data was conducted utilizing the MSstats package in R [16]. All data were normalized by equalizing median intensities, the summary method was Tukey’s median polish, and the maximum quantile for deciding censored missing values was 0.999. For protein abundance analyses, only the top 10 peptide features per protein were considered. All R code used for this study is available on GitHub (see Data Availability).

Principal component analysis. Principal component analyses were performed on log_2_fold-change data matrices indicated using the princomp function in R, with any rows containing NA or infinity values omitted.

Gene Ontology (GO) analysis. Gene Ontology (GO) analysis was conducted using a hypergeometric test with the dhyper function in R. Gene ontologies annotations were downloaded from UniProt and GO definitions from the Gene Ontology Resource on 18 February 2021 [17,18]. The test set was comprised of proteins significantly increasing or decreasing (i.e., |log_2_fold-change| > 1 and adjusted *p*-value < 0.05, excluding infinity values) in the comparison of interest, and the background set was all proteins quantified in the comparison of interest. Enrichment tests were performed for any GO term that had at least 2 overlapping proteins in the test set. Proteins identified by peptides that were not unique to a single protein sequence were excluded from this analysis.

Kinase activity analysis. Kinase activity analysis was performed with the KSEA package in R using log_2_fold-change values to rank phosphorylation sites and using the ProtMapper database of kinase-substrate interactions [19,20]. Only kinase-substrate interactions with a belief score of 1 were used for this analysis. Phosphorylation sites identified by peptides that were not unique to a single protein sequence and phosphorylation sites detected on multiply phosphorylated peptides were excluded from this analysis.

Sequence motif analysis. The Motif-X algorithm in the MoMo Suite was used for sequence motif enrichment of residues flanking phosphorylation sites [21,22]. Flanking residues for phosphorylation sites at each time point were extracted in R. The test set was comprised of sequences surrounding phosphorylation sites that significantly increased or decreased at each time point relative to control, and the background set was comprised of sequences surrounding all phosphorylation sites quantified at each time point. Phosphorylation sites identified by peptides that were not unique to a single protein sequence, phosphorylation sites detected on multiply phosphorylated peptides, and phosphorylation sites with less than 5 amino acids on either side were excluded from this analysis.

## 3. Results

### 3.1. Quantification of Proteome and Phosphoproteome Responses to RIG-I Activation

**Figure 1:**
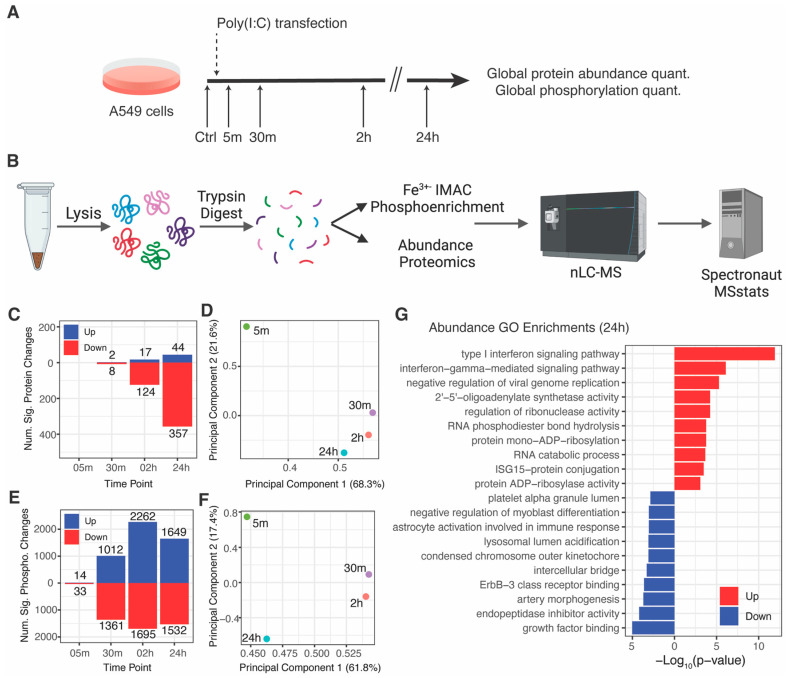
Proteome and phosphoproteome analysis of RLR pathway activation: (**A**) overview of the experimental workflow; (**B**) experimental diagram of MS-based proteomics workflows (created with Biorender.com) (**C**) significantly increased and decreased proteins per time point (|log_2_fold-change| > 1 and adjusted *p*-value < 0.05); (**D**) principal component analysis of protein log_2_fold-change profiles; all proteins were considered except for proteins with missing and infinity values; (**E**) significantly increased and decreased phosphorylation sites per time point (|log_2_fold-change| > 1 and adjusted *p*-value < 0.05); (**F**) principal component analysis of phosphorylation site log_2_fold-change profiles; all phosphorylation sites were considered except for those with missing and infinity values; (**G**) top 10 enriched gene ontology terms for significantly increased and decreased proteins at the 24-h time point.

We applied mass spectrometry-based proteomics to quantify changes in protein and phosphorylation site abundance in A549 human lung epithelial cells in response to RLR pathway activation by poly(I:C) transfection. A549 cells were transfected with poly(I:C) in biological triplicate and harvested at 5 min, 30 min, 2 h, and 24 h post-transfection (Figure 1A,B). All time points were compared to an untransfected control harvested at the 5-min time point. We quantified a total of 5738 protein groups and 14,047 phosphorylation site groups, with an average of 5489 proteins and 11,811 phosphorylation sites quantified per sample (Figure S1, Tables S1 and S2). Throughout this study, we considered changes with |log_2_fold-change| > 1 and adjusted *p*-value < 0.05 to be significant. Most protein changes were observed at least 2 h post-transfection, while substantial phosphorylation changes were observed as early as 30 min post-transfection (Figure 1C,E). Significant increases in many ISGs were observed at 24 h, but the number of significantly decreasing proteins was substantially greater than the number of significantly increasing proteins.

We next performed principal component analysis (PCA) of the protein and phosphorylation site log_2_fold-change profiles for each time point compared to the untransfected control (Figure 1D,F). At the protein level, PC1 and PC2 accounted for 89.9% of the variance and primarily separated the 5-min time point from all others. At the phosphorylation site level, PC1 represented 61.8% of the variance and separated the 5-min and 24-h time points from the 30-min and 2-h time points, while PC2 accounted for 17.4% of the variance and separated the 5-min and 24-h time points from each other. PC1 is consistent with the number of significantly changing phosphorylation sites, which peak at the 2-h time point then decrease at 24 h, and is consistent with negative regulation of the pathway that eventually returns to baseline in the absence of further stimulus.

We next performed an enrichment analysis for gene ontology (GO) terms enriched in proteins that were significantly increased or decreased at each time point (Figure 1G, Table S3). No terms were enriched with an adjusted *p*-value < 0.05 at the 5-min and 30-min time points. Most significantly enriched terms were observed in the 24-h time point and were largely related to increases in proteins involved in type-I IFN response such as OAS1, ISG15, IRF2, STAT2, IRF9, ISG20, SAMHD1, and OAS3. Several processes were enriched amongst significantly decreasing proteins, including lysosomal lumen acidification and endopeptidase inhibitor activity. Many viruses utilize lysosomal acidification to trigger conformational changes that facilitate viral entry, and secreted endopeptidase inhibitors have been described to inhibit viral infection by preventing viral envelope maturation and viral entry [23]. While we observed a significant decrease in endopeptidase inhibitors SPIT2, A2MG, CYTC, FETUA, ITIH2, PZP, these proteins are secreted from the cell and our analysis was restricted to cellular lysates. It is possible that a decrease was observed because innate immune pathway activation induced their release into the extracellular space.

### 3.2. Kinase Activity Analysis Reveals a Decrease in Cellular Kinase Activities in Response to RLR Pathway Activation

**Figure 2:**
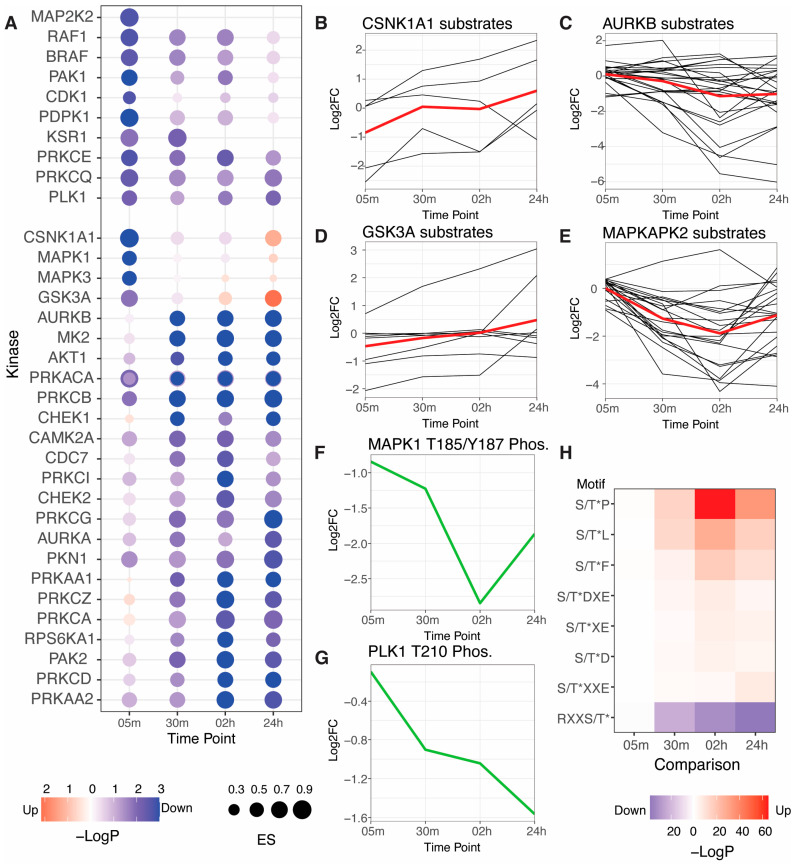
Kinase activity analysis of RLR pathway activation: (**A**) kinase activity changes predicted by the KSEA algorithm based on ProtMapper kinase-substrate interactions [19,20]; node fill color indicates the −log_10_(*p*-value) and node size indicates the KSEA enrichment score (ES); (**B**–**E**) log_2_fold-change plots for individual phosphorylation sites grouped by their annotated upstream kinases: CSNK1A1 substrates (**B**), AURKB substrates (**C**), GSK3A substrates (**D**), and MAPKAPK2 substrates (**E**); black lines indicate individual phosphorylation site profiles; red lines indicate the mean log_2_fold-change profile; (**F**,**G**) log_2_fold-change profiles of phosphorylation sites associated with kinase activity for MAPK1/ERK2 (**F**) and PLK1 (**G**); (**H**) sequence motifs significantly enriched by the Motif-X algorithm; tile color indicates −log_10_(*p*-value).

Kinase activities can be inferred from phosphoproteomics data by comparing it to robust kinase-substrate interaction data. We performed a kinase activity analysis with the KSEA algorithm and using a database of kinase-substrate interactions compiled from six databases (Figure 2A, Table S4) [19,20]. Of kinase activities that increased or decreased with an adjusted *p*-value < 0.05, most kinases were predicted to decrease in activity in response to poly(I:C) transfection. Exceptions were GSK3A, CSNK1A1, MAPK1/ERK2, and MAPK3/ERK1. Kinase activity changes generally agreed with the individual log_2_fold-change profiles of kinase substrates (Figure 2B–E). As KSEA is a rank-based statistical test, we do not expect KSEA *p*-values to precisely reflect the phosphorylation site log2fold-change profiles. We present the phosphorylation site profiles to illustrate the complexity of the data, the trends that can be observed for each kinase, and how well the underlying data corroborates the findings from KSEA analysis. We detected phosphorylation sites on kinases that are reflective of kinase activity for PLK1 and MAPK1/ERK2. PLK1 T210 phosphorylation decreased throughout the time course, in agreement with its kinase activity prediction. MAPK1/ERK2 T185/Y187 dual phosphorylation also decreased throughout the time course, reaching a nadir at the 2-h time point, while kinase activity analysis predicted a weak increase at 24 h.

While most significantly changing kinase activities decreased in response to poly(I:C) transfection, the number of individual phosphorylation sites significantly increasing and decreasing was roughly similar at each time point (Figure 1D, Table S5). Kinase-substrate interaction databases are biased towards a small number of well-studied kinases, so we performed a sequence motif enrichment analysis of residues surrounding phosphorylation sites quantified using the Motif-X algorithm that was independent of any prior information [22]. Motif enrichment revealed a significant enrichment of three types of motifs amongst significantly increased phosphorylation sites: proline-directed motifs, which are common among cyclin-dependent kinases (CDKs) involved in cell cycle regulation and mitogen-activated protein kinases (MAPKs) involved in extracellular signal transduction; acidophilic motifs, which include casein kinases and GSK3A and is consistent with kinase activity predictions for CSNK1A1 and GSK3A; and hydrophobic motifs that are found in many kinase families including IKKs that regulate innate immune pathways (Figure 2H) [24]. Only one motif was significantly enriched amongst significantly decreasing phosphorylation sites: a basophilic motif, which is the most common motif among kinases.

### 3.3. Kinase Inhibitors Targeting ATM, ATR, PLK1, and AMPKA1 Inhibit ISG Induction in Response to RLR Activation

**Figure 3:**
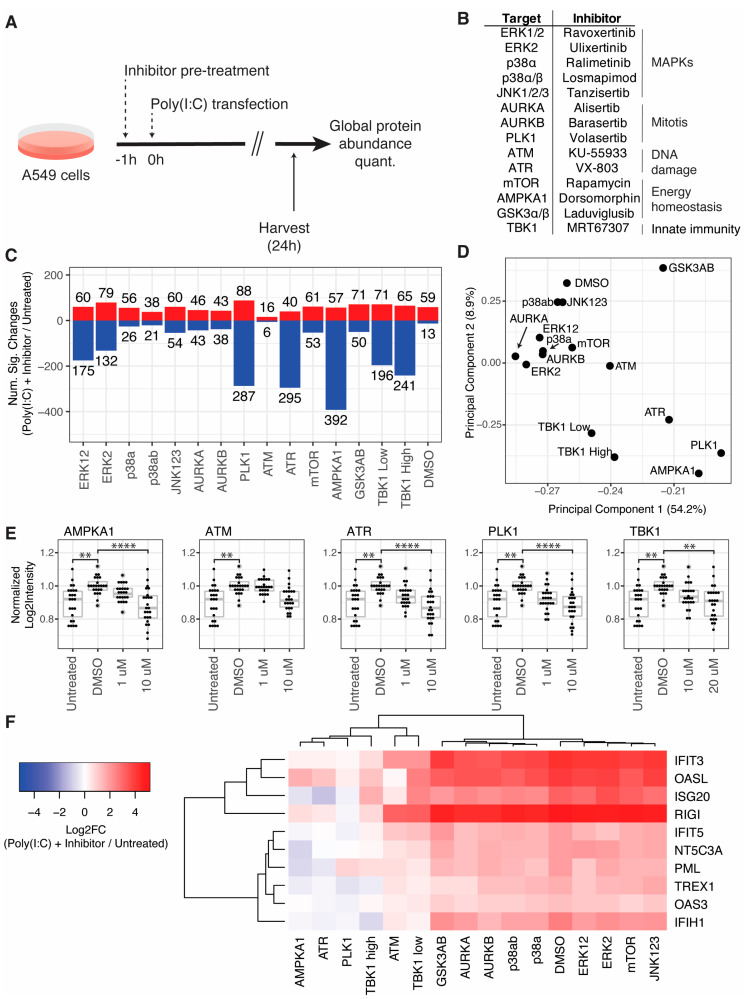
Screen for kinase inhibitors that regulate ISG induction in response to RLR pathway activation: (**A**) overview of the experimental workflow; (**B**) table of kinase inhibitor targets, inhibitor names, grouped by cellular processes; (**C**) number of significantly increasing (red) and decreasing (blue) proteins for cells treated with each kinase inhibitor and transfected with poly(I:C) compared to untreated cells (**D**) principal component analysis of log_2_fold-change profiles for same comparisons in panel (**C**); (**E**) dot and box plots of log_2_Intensities of ISGs with antiviral activity (from [3]) in untreated cells or in response to poly(I:C) transfection and DMSO or inhibitor treatment; each dot represents an ISG; log_2_intensity values were averaged across replicates and normalized to the DMSO condition; proteins with missing values in any conditions indicated were excluded; significance testing was performed by a pairwise *t*-test with Bonferroni correction, ** adjusted *p*-value < 5 × 10^−3^, **** adjusted *p*-value < 5 × 10^−5^; (**F**) log_2_fold-change heatmap of ISGs with antiviral activity for same comparisons in panel (**C**); ISGs with infinite or missing values in any condition were excluded.

To understand crosstalk between cellular pathways and the initiation of innate immune pathways, we carried out a small-scale screen of kinase inhibitors in the context of RLR activation by poly(I:C) transfection (Figure 3A). We selected a panel of fourteen kinase inhibitors targeting kinases that are frequently regulated in viral infections and that span a broad range of cellular activities, including MAPK pathways, mitosis, DNA damage, and energy homeostasis (Figure 3B). A TBK1/IKKε inhibitor was included as a positive control at two concentrations (10 and 20 µM). A549 cells were pretreated with inhibitors at 10 µM or DMSO for 1 h in triplicate, transfected with poly(I:C), and then harvested for quantitative proteome analysis 24 h post-transfection. All samples were compared to an untransfected control that was harvested at the 24-h time point. Inhibitor pretreatments affected the number of significantly changing proteins in response to poly(I:C) transfection (Figure 3C, Table S6). For example, AMPKA1 inhibitor pretreatment resulted in 392 proteins decreasing, compared to 13 proteins decreasing with DMSO pretreatment. We note that the DMSO pretreatment condition is analogous to the 24-h time point assessed in the time course described in Section 3.1 but resulted in a substantially different number of significantly decreasing proteins. The transfection reagent used for these two experiments was different and may account for some of these differences.

Principal component analysis of log_2_fold-change profiles revealed that PC1 and PC2 separated inhibitors targeting ATM, ATR, PLK1, AMPKA1, and TBK1 from all other inhibitors tested and DMSO (Figure 3D). The RLR pathway results in the production of type-I IFN, which induces the expression of hundreds of ISGs. Thus, we extracted the log_2_intensity values of ISGs with documented antiviral activity (from [3]). We compared the intensities of the ISGs in untreated cells to cells transfected with poly(I:C) and treated with DMSO or two concentrations of each of these inhibitors (Figure 3E). Inhibitors targeting AMPKA1, ATR, PLK1, and TBK1 significantly reduced ISG abundance compared to DMSO treatment at the higher concentrations tested. Treatment with an ATM inhibitor reduced ISGs with an adjusted *p*-value of 0.0625. All of these inhibitors except the ATM inhibitor exhibited a dose-dependent reduction in ISGs levels. We next extracted log_2_fold-change profiles for cells transfected with poly(I:C) and treated with DMSO or inhibitors compared to untreated cells for ISGs with antiviral activity (from [3]). We excluded ISGs with non-finite log_2_fold-change values in any condition, which excluded many common ISGs that were observed with positive infinity values due to low basal expression in the untreated condition. Nevertheless, we quantified log_2_fold-change profiles for ten ISGs across all conditions and found that, in agreement with PCA clustering, inhibitors targeting ATM, ATR, PLK1, AMPKA1, and TBK1 reduced the induction of these ISGs (Figure 3E). An ATM inhibitor reduced ISGs to levels similar to the lower dose (10 µM) of TBK1 inhibitor, while AMPKA1 and ATR inhibitors reduced ISGs to levels similar to the higher dose (20 µM) of TBK1 inhibitor, and a PLK1 inhibitor reduced ISGs more than any inhibitors tested. We tested the impact of all inhibitor treatments on cell viability and found that AMPKA1 and TBK1 inhibitors reduced viability, while GSK3AB and mTOR inhibitors increased viability readouts, likely due to an Increase in cell proliferation (Figure S3A).

To understand the impact of kinase inhibitors on cellular processes, we performed GO enrichment analysis of log_2_fold-change profiles for cells pretreated with inhibitors and transfected with poly(I:C) compared to cells pretreated with DMSO and transfected with poly(I:C) (Figure S4, Table S7). The most significantly enriched GO terms reflected downregulation of proteins in the type-I IFN pathway in cells pretreated with inhibitors targeting ATM, ATR, AMPKA1, PLK1, and TBK1, and these processes drove most of the clustering of kinase inhibitors by GO processes. GO analysis revealed other cellular processes impacted by inhibitors. A cluster of GO terms related to low-density lipoprotein particles and cholesterol metabolism were upregulated in cells pretreated with inhibitors targeting AMPKA1, PLK1, and p38a. Similarly, pretreatment of cells with inhibitors targeting TBK1, ERK2, and mTOR led to a downregulation of proteins related to ubiquitination processes.

### 3.4. Genetic Kinase Depletion Does Not Confirm Effects of Kinase Inhibitors in Response to RLR or IFN Pathway Activation

**Figure 4:**
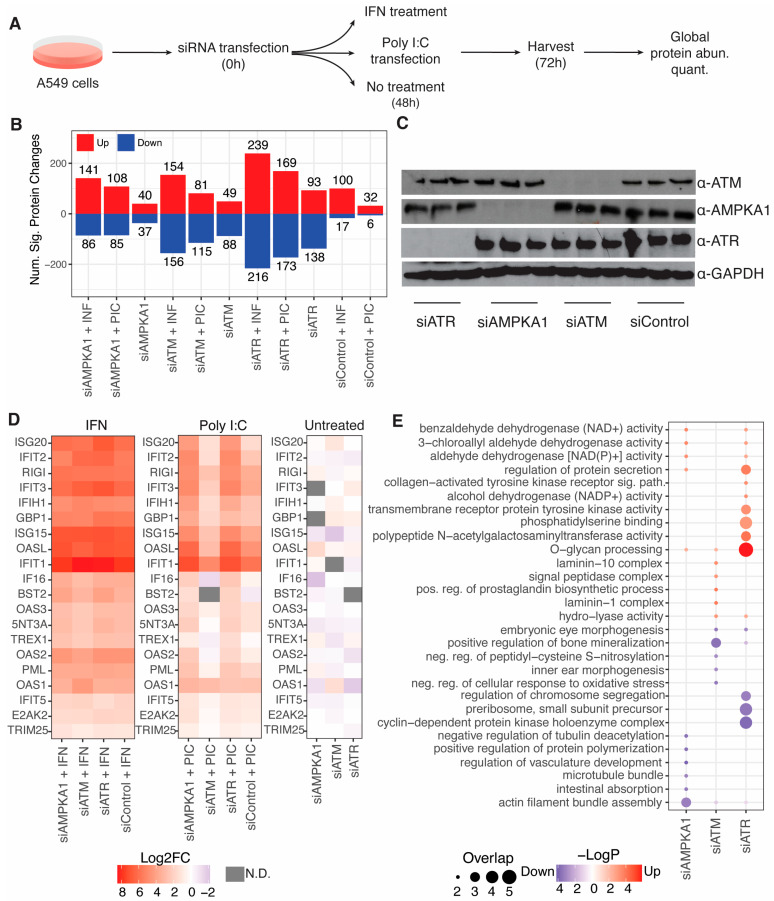
Genetic analysis of kinase regulation of RLR and IFN pathways: (**A**) overview of the experimental workflow; (**B**) number of significantly increasing and decreasing proteins for cells transfected with each siRNA and transfected with poly(I:C) compared to non-targeting control siRNA transfected cells that were not transfected with poly(I:C); (**C**) Western blot verification of siRNA targets depletion; (**D**) log_2_fold-change heatmaps of ISGs with antiviral activity; (**E**) GO enrichment analysis of cells transfected with kinase-targeting siRNAs compared to non-targeting control in cells not transfected with poly(I:C).

Kinase inhibitors can have off-target effects, so we sought to confirm our findings regarding kinase inhibitors that inhibit ISG induction by measuring ISG induction in cells depleted for kinases by siRNA (Figure 4A). A549 cells were transfected in triplicate with siRNA pools targeting ATM, ATR, PLK1, AMPKA1, or a non-targeting control pool. Cells transfected with siRNA targeting PLK1 died and were thus excluded from further analysis. Then, 48 h after siRNA transfection, each population of siRNA-transfected cells was split into three populations, which were then either transfected with poly(I:C), treated with IFN, or left untreated. Cells were harvested 24 h after the second treatment (i.e., 72 h after siRNA transfection) for quantitative proteome analysis. All conditions were compared to untreated cells transfected with a non-targeting control siRNA pool. IFN treatment induced the most significant protein changes for each siRNA condition, followed by poly(I:C) transfection, and no treatment (Figure 4B, Table S8). We confirmed siRNA depletion of kinase targets by Western blotting (Figure 4C).

We next extracted log_2_fold-change profiles for ISGs with antiviral activity, filtering out proteins with non-finite log_2_fold-change values in any condition (Figure 4D). The ISG log_2_fold-change profiles for cells treated with IFN were virtually indistinguishable; all siRNA conditions induced ISGs to similar levels. For cells transfected with poly(I:C), however, siRNA pools targeting AMPKA1 and ATR resulted in a greater induction of ISGs compared to control, while the ISG profile for cells transfected with an ATM-targeting siRNA pool was not significantly different from non-targeting control. These observations contrast with our observations with kinase inhibitors described above, where inhibitors targeting these kinases substantially reduced ISG induction. Gene ontology enrichment analysis of proteins significantly changing in response to kinase depletion in untreated cells compared to non-targeting control siRNA transfection in untreated cells revealed processes perturbed by kinase depletion (Figure 4E, Table S9). ATR depletion resulted in the strongest GO enrichments; its depletion increased proteins involved in glycan processing and receptor tyrosine kinase activity and decreased proteins involved in cyclin-dependent protein kinase activity and chromosome segregation. AMPKA1 depletion decreased proteins involved in actin filament bundle assembly, and ATM depletion decreased proteins involved in bone mineralization.

### 3.5. Cellular Kinase Inhibitors Block Innate Immune Signaling by Different Mechanisms

**Figure 5:**
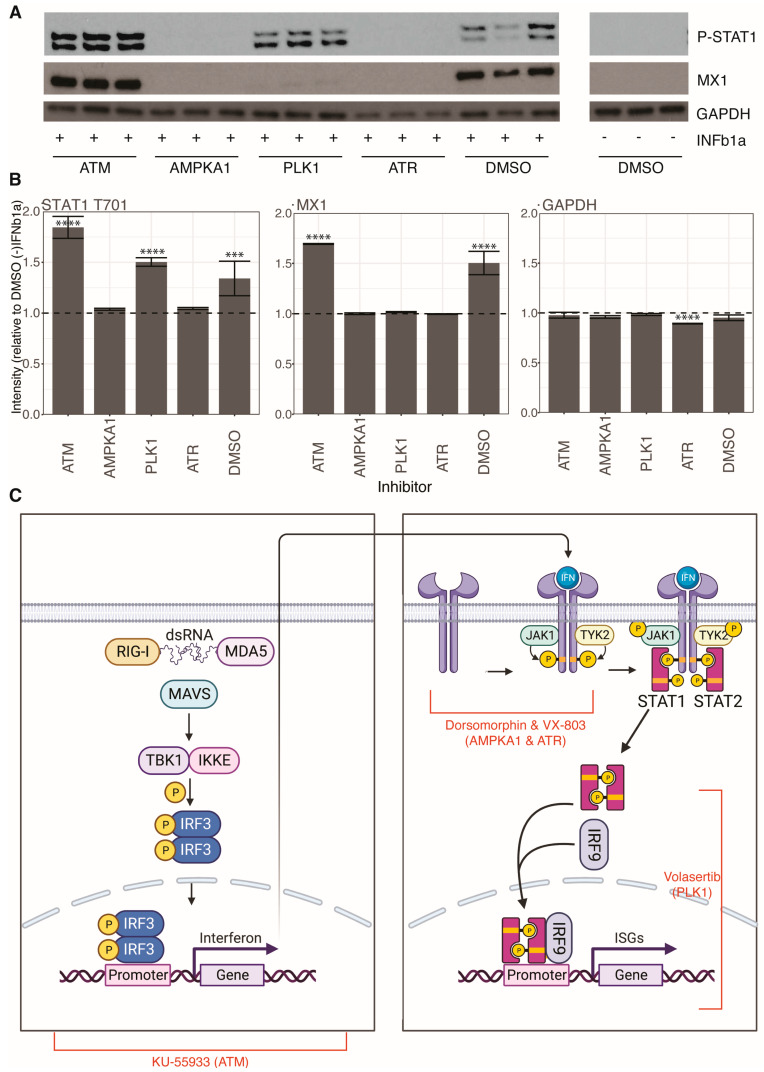
Mapping kinase inhibitor activity in innate immune signaling pathways: (**A**) Western blotting analysis of A549 cells pretreated with kinase inhibitors or DMSO and treated with IFN; (**B**) densitometry analysis of images from panel (**A**) by ImageJ; significance testing by pairwise *t*-tests with Bonferroni correction; *** adj. *p*-value < 5 *×* 10^−4^, **** adj. *p*-value < 5 *×* 10^−5^; (**C**) schematic of the RLR and IFN pathways with kinase inhibitor activity mapped to certain steps in the pathways (created with BioRender.com).

To understand where in the RLR and IFN pathways ATM, ATR, AMPKA1, and PLK1 inhibitors exert their effects, we pretreated cells with kinase inhibitors or DMSO for 1 h before IFN treatment for 24 h and measured induction of a prototype ISG, MX1, and STAT1 phosphorylation by Western blotting (Figure 5A). ATM inhibition did not significantly impact either MX1 induction or STAT1 phosphorylation compared to DMSO control, suggesting that ATM inhibition antagonizes the RLR signaling pathway but does not impact the IFN response pathway. AMPKA1 and ATR inhibition blocked both MX1 induction and STAT1 phosphorylation, indicating that they antagonize the IFN pathway at a level before STAT1 phosphorylation. PLK1 inhibition did not impact STAT1 phosphorylation, but did inhibit MX1 induction, suggesting that it impacts the IFN pathway at a step after STAT1 phosphorylation, such as nuclear translocation of ISGF3 or inhibition of ISG expression or translation.

## 4. Discussion

In this study, we characterized RLR and IFN pathways with quantitative proteomics and phosphoproteomics approaches. We found that poly(I:C) transfection resulted in modulation of processes that are critical for viral infections, including secreted endopeptidase inhibitor activity and lysosomal lumen acidification. Similarly, kinase activity analysis revealed the poly(I:C) transfection results in a drastic downregulation in many cellular kinase activities. Multiple viruses utilize cellular kinases to promote viral infection. We have previously described how AURKA activity promotes HIV-1 replication, and how p38/MAPK activity promotes SARS-CoV-2 replication [12,13,25]. Validation of these findings in additional cell types may uncover common novel mechanisms by which innate immune pathways downregulate cellular processes to create an environment inhospitable to viral replication.

A small-scale kinase inhibitor screen identified inhibitors of ATM, ATR, AMPKA1, and PLK1 that significantly antagonized innate immune pathways. Our findings on PLK1 are consistent with a report that PLKs are essential components of antiviral pathways [26]. Our findings on ATM and ATR contrast with studies that propose that inhibition of DNA damage pathways leads to an accumulation of damaged DNA that is sensed by cellular PRRs and activates the type-I IFN system [27,28,29]. Notably, our siRNA experiments revealed increased ISG induction in cells transfected with siRNA targeting ATR compared to non-targeting control. This may be a result of the longer time scale for siRNA experiments (i.e., 3 days) that may lead to accumulation of damaged DNA compared to the shorter time scale for kinase inhibitor experiments (i.e., 1 day). Our findings on AMPKA1 are consistent with a report that AMPK promotes innate immunity by modulating STING signaling [30].

Unfortunately, we were unable to confirm the effects of kinase inhibitors on innate immune signaling with siRNA depletion of the cognate kinases. It is possible that the effects we observed are due to off-target effects of inhibitors. An inhibitor may have activity against a family or class of kinases that is not validated by individual siRNA kinase depletion. It is also possible that the extended time scale of siRNA experiments leads to disruption of negative feedback mechanisms that rewire cellular pathways to compensate for target depletion. For example, kinase inhibition in the context of cancer treatment frequently leads to pathway rewiring, treatment resistance, and treatment failure [31]. Regardless, the inhibitors assayed in this study had clear and significant effects on innate immune signaling. We mapped the activity of each inhibitor to specific steps in RLR and IFN signaling pathway and found that they impact the immune response at different levels. Determining the specific targets and mechanisms by which these inhibitors antagonize innate immune signaling can illuminate novel mechanisms of control.

## Data Availability

Raw mass spectrometry data and Spectronaut report files have been deposited to the ProteomeXchange Consortium via the PRIDE partner repository with the dataset identifier PXD041369 [32,33]. All R code for analyses described in this study are available on GitHub at https://github.com/jrjohns1/Kurland-et-al/.

## Supplementary Materials

The following supporting information can be downloaded at: https://www.mdpi.com/article/10.3390/v15051112/s1, Figure S1: Quality control plots related to RLR pathway activation time course proteomics, Figure S2: Quality control plots related to kinase inhibitor screen proteomics, Figure S3: Impact of kinase inhibitors on cell viability, Figure S4: GO enrichment analysis of kinase inhibitors, Figure S5: Quality control plots related to kinase siRNA depletion proteomics; Table S1: MSstats results related to RLR pathway activation time course proteomics, Table S2: MSstats results related to RLR pathway activation time course phosphoproteomics, Table S3: Gene ontology enrichment analysis of protein increased or decreased by RLR pathway activation, Table S4: Kinase activity analysis of RLR pathway activation time course phosphoproteomics data, Table S5: Motif-x analysis of RLR pathway activation time course phosphoproteomics data, Table S6: MSstats results related to kinase inhibitor screening proteomics, Table S7: Gene ontology enrichment analysis of kinase inhibitor screening proteomics data, Table S8: MSstats results related to kinase siRNA depletion proteomics, Table S9: Gene ontology enrichment analysis of kinase siRNA depletion proteomics data.
