# Supplementary material for: A Chemical Proteomics Approach to Discover Regulators of Innate Immune Signaling
Source: Viruses. 2023 May 3;15(5):1112. doi: 10.3390/v15051112 (PMC10223003; doi:10.3390/v15051112)
Supplement: Supplementary file 1 [file viruses-15-01112-s001.zip › Figure S1.pdf]

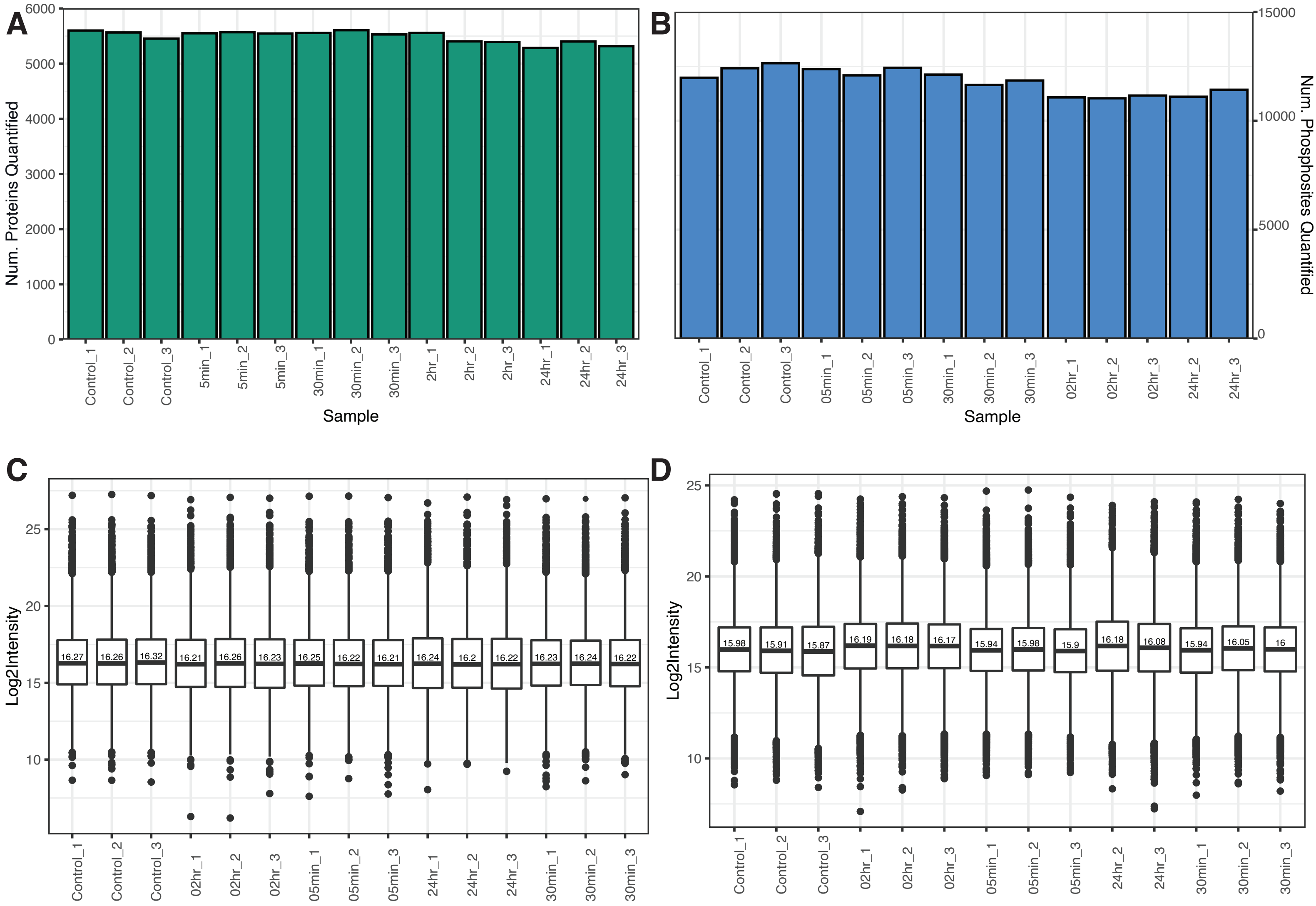

Figure S1. Quality control plots related to RLR pathway activation time course: (a) Number of proteins quantified per sample; (b) number of phosphorylation sites quantified per sample; (c) box plots of intensity distributions of proteins in each sample; (d) box plots of itensity distributions of phosphorylation sites in each sample. The box plots are in the Tukey style, central line indicates median, the hinges of the box indicate the 25th and 75th quartiles of data. The whiskers indicate the largest/smallest values no further than 1.5 * IQR from the closest hinge (IQR inter-quartile range).
