# Supplementary material for: A Chemical Proteomics Approach to Discover Regulators of Innate Immune Signaling
Source: Viruses. 2023 May 3;15(5):1112. doi: 10.3390/v15051112 (PMC10223003; doi:10.3390/v15051112)
Supplement: Supplementary file 1 [file viruses-15-01112-s001.zip › Figure S3.pdf]

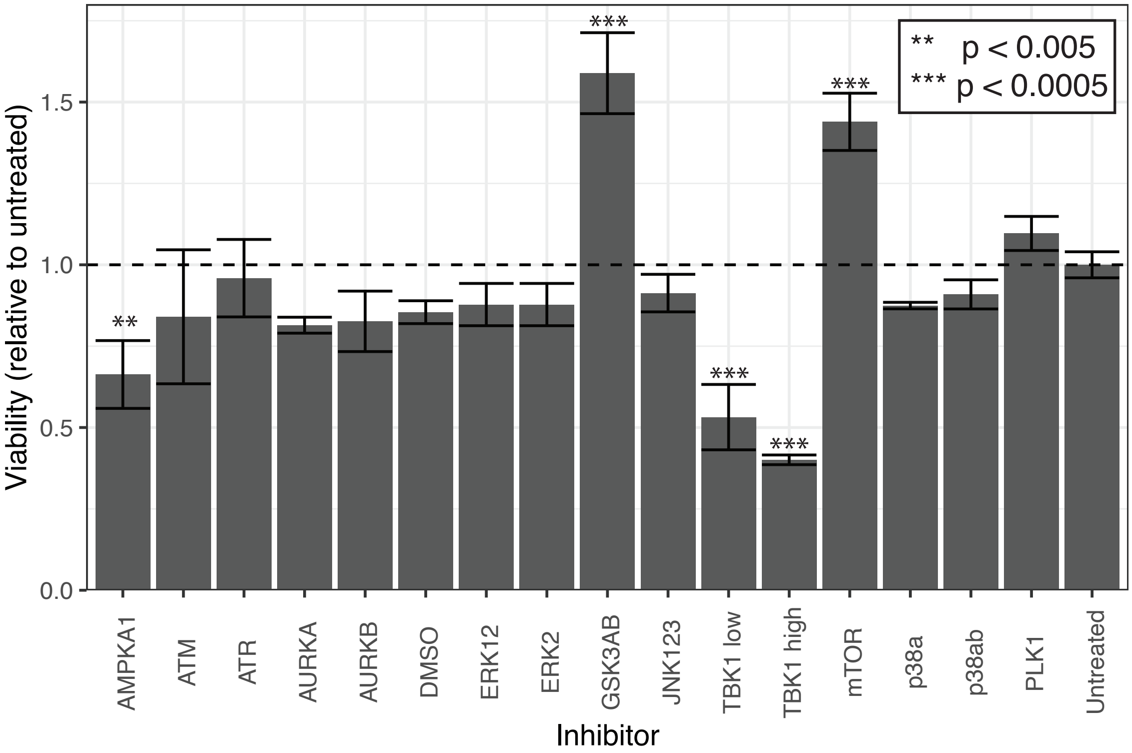

Figure S3. Impact of kinase inhibitors on cell viability: A549 cells were treated with inhibitors (indicated by target) for 24 hours and assayed with Cell TiterGlo viability system. Viability is quantified relative to untreated cells. Error bars indicate standard deviation of three biological replicates.
