# Supplementary material for: A Chemical Proteomics Approach to Discover Regulators of Innate Immune Signaling
Source: Viruses. 2023 May 3;15(5):1112. doi: 10.3390/v15051112 (PMC10223003; doi:10.3390/v15051112)
Supplement: Supplementary file 1 [file viruses-15-01112-s001.zip › Figure S4.pdf]

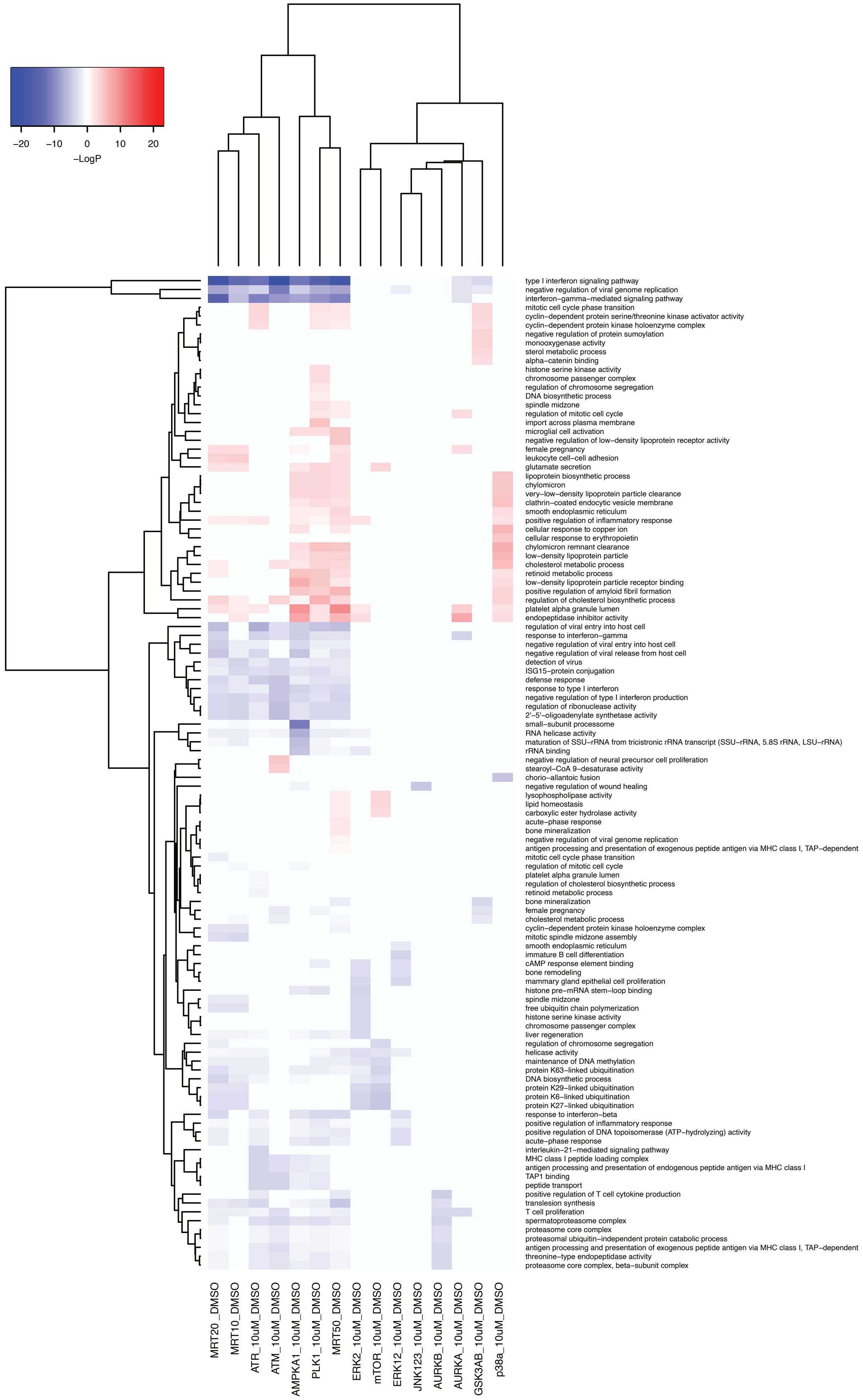

Figure S4. GO enrichment analysis of kinase inhibitors: GO enrichment analysis was performed on log2fold-change profiles for A549 cells pretreated with inhibitors and transfected with poly(I:C) compared to cells pretreated with DMSO and transfected with poly(I:C). Tile fill color indicates the -log10(p-value) of enrichment.
